# Clinical efficacy and tolerability of venetoclax plus rituximab in patients with relapsed or refractory chronic lymphocytic leukemia—a real-world analysis of the Polish Adult Leukemia Study Group

**DOI:** 10.1007/s00277-023-05304-4

**Published:** 2023-07-01

**Authors:** Anita Soboń, Joanna Drozd-Sokołowska, Ewa Paszkiewicz-Kozik, Lidia Popławska, Marta Morawska, Jagoda Tryc-Szponder, Łukasz Bołkun, Justyna Rybka, Katarzyna Pruszczyk, Adrian Juda, Alan Majeranowski, Elżbieta Iskierka-Jażdżewska, Paweł Steckiewicz, Kamil Wdowiak, Bożena Budziszewska, Krzysztof Jamroziak, Iwona Hus, Ewa Lech-Marańda, Bartosz Puła

**Affiliations:** 1 Department of Hematology, Institute of Hematology and Transfusion Medicine, Indira Gandhi Str. 14, 02-776 Warsaw, Poland; 2 Department of Hematology, Transplantation and Internal Medicine, Medical University of Warsaw, Warsaw, Poland; 3 Department of Lymphoid Malignancies, Maria Sklodowska-Curie National Research Institute of Oncology, Warsaw, Poland; 4 Experimental Hematooncology Department, Medical University of Lublin, Lublin, Poland; 5 Hematology Department, St. John’s Cancer Center, Lublin, Poland; 6 Department of Hematology and Bone Marrow Transplantation, Poznan University of Medical Sciences, Poznań, Poland; 7 Department of Hematology, Medical University of Białystok, Białystok, Poland; 8 Department of Hematology, Blood Neoplasms and Bone Marrow Transplantation, Wroclaw Medical University, Wrocław, Poland; 9 Department of Hematology and Bone Marrow Transplantation, Medical University of Lublin, Lublin, Poland; 10 Department of Hematology and Transplantology, Medical University of Gdańsk, Gdańsk, Poland; 11 Department of General Hematology, Medical University of Lodz, Copernicus Memorial Hospital, Lodz, Poland; 12 Department of Hematology, Holy Cross Cancer Center, Kielce, Poland; 13 Department of Internal Diseases and Oncological Chemotherapy Faculty of Medical Sciences, Medical University of Silesia, Katowice, Poland; 14 Department of Hematology, The National Institute of Medicine of the Ministry of Interior and Administration, Warsaw, Poland

**Keywords:** Chronic lymphocytic leukemia, Venetoclax, Rituximab, Therapy

## Abstract

The results of the MURANO trial showed encouraging progression-free survival (PFS) and overall survival (OS) in relapsed/refractory chronic lymphocytic leukemia (RR-CLL) patients treated with venetoclax-rituximab (VEN-R). A retrospective analysis was performed to evaluate the efficacy and safety of VEN-R within the Polish Adult Leukemia Study Group (PALG) centers. The study group included 117 patients with RR-CLL (with early relapse after immunochemotherapy or bearing TP53 aberrations) treated with VEN-R in 2019–2023 outside clinical trials. Patients were treated with a median of 2 (range 1–9) previous lines of therapy. Twenty-two participants were previously treated with BTKi (18.8% out of 117). The median follow-up was 20.3 months (range 0.27–39.1). The overall response rate (ORR) was 95.3% in the group of patients in whom a response to treatment was assessed and 86.3% for all patients. Twenty patients (17.1% out of 117) achieved a complete response (CR), 81 (69.2%) achieved a partial response (PR), and in 5 patients (4.3%), disease progression was noted (assessed as the best response during treatment). The median PFS in the whole cohort was 36.97 (95% CI 24.5, not reached) months, and the median OS was not reached (95% CI 27.03, not reached). Thirty-six patients died during the follow-up, 10 (8.5%; 27.8% of deaths) due to COVID-19 infection. All grade neutropenia (*n* = 87/117, 74.4%; grade 3 or higher *n* = 67/117, 57.3%) was the most common treatment adverse event. Forty-five patients (38.5%) remained on treatment, and twenty-two (18.8%) completed 24 months of therapy, while it was discontinued in fifty cases (42.7%). In this real-world setting of early access in very high-risk RR-CLL patients, the VEN-R regimen was associated with shorter median PFS compared with the results of the MURANO trial. This outcome, however, could be attributed to patients’ exposure to SARS-CoV-2 infection and the aggressive course of the disease as very high-risk patients, after multiple lines of prior therapies, were included in the Polish Ministry of Health reimbursement program.

**Supplementary Information:**

The online version contains supplementary material available at 10.1007/s00277-023-05304-4.

Chronic lymphocytic leukemia (CLL) is the most common leukemia in the adult population, characterized by clonal proliferation and accumulation of mature-looking, immunoincompetent B-lymphocytes in the blood, bone marrow, and lymphoid organs [1]. The clinical course of CLL may be indolent or aggressive, mainly in patients with unfavorable cytogenetic and molecular risk factors [1–3]. Adverse risk factors include the presence of 17p deletion or/and *TP53* mutation associated with the resistance to anti-CD20 antibody-based immunochemotherapy and a propensity to transform into aggressive lymphoma and unmutated *IGHV* genes associated with shorter response to immunochemotherapy [3, 4].

In recent years, CLL treatment options have expanded to include new anti-CD20 antibodies B, cell receptor (BCR) inhibitors, and BCL2 antagonist venetoclax [2, 3, 5–8]. The anti-apoptotic protein BCL2 is a key regulator of the intrinsic apoptotic pathway and has been found to be overexpressed in CLL cells. Venetoclax acts independently of *TP53* and was first approved for marketing in the European Union in 2016 in patients with a 17p deletion/*TP53* mutation in whom treatment with immunochemotherapy and a BCR inhibitor has failed [3, 8, 9]. Marketing approval was based on the phase I/II study results involving 116 patients with relapsed/refractory lymphocytic leukemia (RR-CLL). In this study, a response was achieved in 80% of patients, of which 20% achieved complete remission (CR). The treatment was also effective in patients with 17p deletion [8]. In March 2018, the results of the phase III MURANO trial were published, demonstrating the superiority of the combination of venetoclax and rituximab (VEN-R) over the bendamustine and rituximab (BR) in terms of overall response rate (ORR; 90% vs. 72%), progression-free survival (PFS), and overall survival (OS) in relapsed and refractory CLL patients [10, 11]. The promising outcomes of this trial have resulted in the approval of VEN-R in the therapy of patients with RR-CLL in the European Union and the USA [12]. However, it should be noted that the results of registration studies often do not correspond with the data from real-world setting. Among the reasons for such differences are the strict inclusion and exclusion criteria for clinical trials and the different levels of experience of centers providing treatment with a given therapy.

Herein, we report the initial results of the retrospective analysis of the efficacy and safety of the VEN-R protocol in patients with RR-CLL treated in Polish Adult Leukemia Group (PALG) sites. Due to the inclusion criteria for the reimbursement program, the analyzed group should be regarded as a high-risk cohort.

## Material and methods

### Study population

In the retrospective analysis, patients treated with VEN-R within the Ministry of Health reimbursement program in Poland initiated in 2018 were included. At that time, inclusion criteria for VEN-R treatment in the program were as follows: (1) diagnosis of RR-CLL; (2) age 18 years or older; (3) WHO performance status 0–1; (4) no contraindications to VEN-R, as described in the relevant Summary of Product Characteristics (SPC); (5) presence of *TP53* mutation or 17p deletion; or (6) in the absence of del17p/mut *TP53* CLL resistance after at least 1 line of immunochemotherapy (no response or relapse within 6 months from the end of treatment in RR-CLL patients) or early relapse within 6–24 months after the end of the first-line treatment. The study was conducted following the provisions of the Declaration of Helsinki and the International Conference on Harmonization Guidelines for Good Clinical Practice.

All the patients received treatment with VEN-R according to SPC [16]. Indications to therapy and response evaluation were based on the 2018 International Workshop on Chronic Lymphocytic Leukemia (iwCLL) criteria [13]. The drug program requires (after the dose adjustment period) a peripheral blood count with a smear every month and biochemical tests every 3 months at a minimum. The first assessment of response to treatment should be performed after 3 months of therapy, every 3 months for the first year of therapy, and then every 6 months. The choice of imaging studies as part of the radiological assessment is at the treating physician’s discretion (possible combinations include abdominal ultrasound and chest X-ray or CT or MRI). Usually (unless there were exceptional circumstances requiring the patient to be examined sooner), follow-up visits were coordinated with the above schedule.

All patients treated according to the schedule at the participating centers at the time of closure of the database were included. The overall response rate (ORR) was defined as the proportion of patients achieving a CR or PR. PFS and OS were calculated from the date of initiation of VEN-R treatment until progression or death or death from any cause, respectively. Adverse events (AE) during treatment were graded based on the National Cancer Institute Common Terminology Criteria for Adverse Events Assessment, version 4.

### Statistical analysis

The data were analyzed using Statistica 13 (Dell Inc., StatSoft Polska Sp. z o.o., Kraków, Poland, Graph Pad Prism 9 (LA Jolla, CA, USA) and SAS software (SAS Institute Inc., Cary, NC, USA). For univariate analysis, we have used the Mann–Whitney *U*, Fisher’s exact, and chi-square tests, as appropriate. The level of statistical significance was set at *p* < 0.05. The hazard ratio (HR) and 95% confidence interval (95% CI) were calculated in each case. A *p*-value less than 0.05 was considered statistically significant.

## Results

### Baseline characteristics

Clinical data of 117 patients with RR-CLL treated with VEN-R were collected (Table 1). Median patients’ age upon initiation of therapy was 67 years (range 33–84 years). Seventy-two (61.5% out of 117) patients were men. Patients were treated with a median of 2 (range 1–9) previous lines of therapy. Twenty-two participants were previously treated with BTKi (18.8%). Of the above-mentioned patients, treatment was terminated in 15 (*n* = 15/22, 68.2%) cases due to disease progression and in 5 (22.7%) due to drug intolerance. Only three (2.6%) people were previously treated with PI3K inhibitors in the entire cohort. The median performance status of patients as assessed by the Eastern Cooperative Oncology Group (ECOG) score was one, while the median Cumulative Illness Rating Scale (CIRS) was 6 (range 2–16). Twenty-five out of 104 patients tested (24%) had confirmed 17p deletion by fluorescent in situ hybridization (FISH), and 13 (16.5%) had confirmed *TP53* mutation assessed using the Sanger sequencing method (tested in 79 patients). The median follow-up was 20.3 months (range 0.27–39.1).

**Table 1:** Patients’ clinicopathological characteristics

Parameter	All patients
Number of patients	117
Observation time (median (range)) (months)	20.3 (0.27–39.1)
Age (median (range)) (years)	67 (33–84)
Sex (*n*, %)	
Male	72 (61.5)
Female	45 (38.5)
Rai classification (*n*, %)	
0	11 (9.4)
1	23 (19.7)
2	45 (38.5)
3	13 (11.1)
4	21 (17.9)
ND	4 (3.4)
ECOG performance status (*n*, %)	
0	10 (8.5)
1	85 (72.6)
2	16 (13.7)
3	1 (0.9)
ND	5 (4.3)
Cumulative Illness Rating Scale score (median (range))	6 (2–16)
Cytogenetic risk factors (*n*, %)	
Del17p ( +)	25 (21.4)
Del17p ( −)	79 (67.5)
Del17p (ND)	13 (11.1)
* TP53* mutation ( +)	13 (11.1)
* TP53* mutation ( −)	66 (56.4)
* TP53* mutation (ND)	38 (32.5)
del13 ( +)	42 (35.9)
del13 ( −)	40 (34.2)
del13 (ND)	35 (29.9)
ATM ( +)	33 (28.2)
ATM ( −)	56 (47.9)
ATM (ND)	28 (23.9)
tri12 ( +)	10 (8.6)
tri12 ( −)	68 (58.1)
tri12 (ND)	39 (33.3)
Lines of previous treatments (median (range))	2 (1–9)
WBC (median (range)) (G/l)	32.79 (2.71–239.92)
HGB (median (range)) (g/dl)	11.0 (7.2–16.1)
PLT (median (range)) (G/l)	126 (9–344)
History of autoimmune hemolytic anemia	16 (13.7)
History of autoimmune thrombocytopenia	4 (3.4)
Previous therapies (*n*, %)	
Fludarabine, cyclophosphamide, rituximab (FCR)	61 (52.1)
Bendamustine + CD20 antibody	59 (50.4)
Chlorambucil + CD20 antibody	28 (23.9)
BTK inhibitor	22 (18.8)
High-dose methylprednisolone (HDMP)	21 (17.9)
PI3K inhibitor	3 (2.6)
Rituximab exposed (*n*, %)	107 (91.5)
Obinutuzumab exposed (*n*, %)	9 (7.7)

*ECOG*, Eastern Cooperative Oncology Group; *HGB*, hemoglobin; *PLT*, platelets; *WBC*, white blood count, *ND*, no data

### Efficacy

**Fig. 1:**
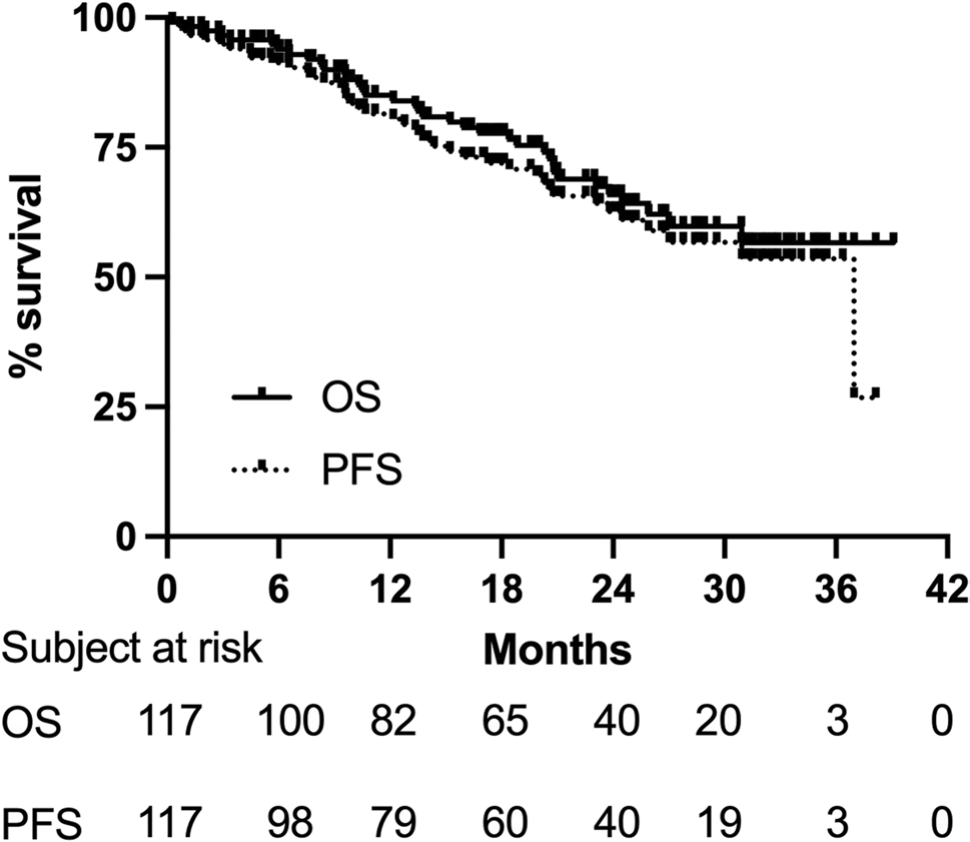
Kaplan–Meier Survival curves of progression-free survival (PFS) and overall survival (OS) of CLL patients treated with venetoclax and rituximab

In the whole cohort 86.3% ORR was noted. For patients in whom a response to treatment was assessed, it was 95.3%. Twenty patients (17.1% out of 117) achieved CR, and 81 (69.2%) achieved PR, while in 5 patients (4.3%), disease progression was noted. In 11 (9.4%) patients, treatment efficacy was not assessed. CR or PR was achieved in all evaluated patients with 17p deletion (*n* = 23) or *TP﻿5﻿3 *mutation (*n* = 12). The median PFS in the whole cohort was 36.97 (95% CI 24.5, not reached) months, and the median OS was not reached (95% CI 27.03, not reached) (Fig. 1). In patients previously treated with ibrutinib, the median PFS was 36.97 months (17.27, not reached), and the median OS was not reached (16.83, not reached). In the analysis, we noted that a creatinine level greater than or equal to 1.3 mg/dl was an adverse prognostic factor on overall survival (OS) for patients treated with venetoclax and rituximab. Since there was only one parameter statistically significant in univariate analysis, we did not perform multivariate analysis. None of the other analyzed clinical or laboratory parameters influenced ORR, PFS, and OS (Table S1, Table S2, and Table S3). During the follow-up, six cases of Richter transformation were diagnosed (5.1%), and 36 deaths were recorded.

### Safety analysis

The most common adverse event of VEN-R was neutropenia (all grades *n* = 87/117, 74.4%; grade 3 or higher in 57.3%, *n* = 67/117), whereas anemia of any grade occurred in 59 (50.4%) patients, grade 3/4 in 15 cases (12.8%) (Table 2). Thrombocytopenia was observed in 54 patients (46.2%), with grade 3 in 5 (4.3%) patients and grade 4 in 12 (10.3%). Febrile neutropenia was reported in 8 patients (6.8%) and pneumonia in 28 (23.9%), including 20 (17.1%, 71.4% of all pneumonia) cases of SARS-CoV-2 etiology. Autoimmune hemolytic anemia (AIHA) was noted in 5 patients (4.3%), including three with a history of AIHA. Immune thrombocytopenic purpura (ITP) occurred in 2 patients (1.7%); one patient had a previous history of ITP. Five patients had grade 3 or 4 diarrhea (4.3%). The uncommon treatment complications that have been reported included stroke (*n* = 1), edema and ascites (*n* = 1), and exacerbation of chronic heart failure to New York Heart Association (NYHA) functional class IV with moderately impaired left ventricular systolic function (EF 45%) (*n* = 1).

**Table 2:** Adverse events according to Common Terminology Criteria for Adverse Events (CTCAE) observed during therapy with VEN-R among patients with relapsed and refractory chronic lymphocytic leukemia

Adverse event	Any grade	Grade 3 or 4
Neutropenia	87 (74.4%)	67 (57.3%)
Anemia	59 (50.4%)	15 (12.8%)
Thrombocytopenia	54 (46.2%)	17 (14.5%)
Pneumonia	28 (23.9%)	ND
Pneumonia of SARS-CoV-2 etiology	20 (17.1%)	ND
Pneumonia of other than SARS-CoV-2 etiology	8 (6.8%)	ND
Febrile neutropenia	8 (6.8%)	ND
Autoimmune hemolytic anemia	5 (4.3%)	ND
Diarrhea	ND	5 (4.3%)
Immune thrombocytopenic purpura	2 (1.7%)	ND
Heart failure	1 (0.9%)	ND
Stroke	1 (0.9%)	ND
Edema and ascites	1 (0.9%)	ND

*NA*, not applicable; *ND*, no data

Thirteen disease progressions (11.1%) were noted during the follow-up, including six cases of Richter transformation (5.1%). From the data that we have obtained, it is known that among patients who had disease progression during the VEN-R treatment, five patients received salvage therapy with ibrutinib, two with pirtoburinib as part of a clinical trial, one patient received polatuzumab-bendamustine-rituximab (Pola-BR) regimen, and one patient received R-CHOP in combination with lenalidomide as a salvage treatment. Four patients did not receive another line of therapy due to critical medical condition and/or severe infection at the time of progression diagnosis. There were 36 deaths recorded: 6 (6/36, 16.7%) due to disease progression, 10 (10/36, 27.8%) due to COVID-19 infection, and 6 (6/36, 16.7%) due to infections other than SARS-CoV-2. One patient died due to relapse of another neoplasm (colorectal cancer), and one patient died in pancytopenia during the diagnosis of myelodysplastic syndrome. The cause of death was not specified in twelve cases (12/36, 33.3%). Forty-five patients (38.5% out of 117) remain on treatment, and 22 (18.8%) completed 24 months of therapy, while therapy was discontinued in fifty cases (42.7%). Treatment was discontinued due to patient’s death in 15 cases (15/50, 30%). Nine of the deaths were due to SARS-CoV-2 infection. Other reasons for treatment discontinuation were therapy-related cytopenia (*n* = 8, 16%), disease progression (*n* = 10, 20%), AIHA (*n* = 1, 2%), diarrhea (*n* = 1, 2%), and infection (*n* = 6, 12%). In two cases, treatment discontinuation was due to withdrawal of consent, in one case due to the progression of a second malignancy, and one patient was lost to follow-up. In one case, the patient arbitrarily interrupted the treatment. No data were available on the reasons for treatment discontinuation in four cases.

## Discussion

In this study, we analyzed the efficacy and safety of VEN-R therapy in 117 patients with RR-CLL outside of clinical trials. Unlike patients in the MURANO trial, our study group was a higher risk and more heavily pretreated. Although we noted the high response rate (ORR 95.3% (in patients with assessed response, 86.3% in the whole cohort), compared to 92.3% of ORR (in the whole cohort) in the MURANO trial, it did not translate into long median PFS—which in our study was moderate (36.97 months) comparing to the one observed in the MURANO trial (at a median follow-up of 59.2 months, the median PFS was 53.6 month) [14]. The VEN-R treatment in Poland was, at the time of database closure, only reimbursed in the relapsed and refractory CLL patients with aggressive clinical features—it should be noted that the inclusion criteria for VEN-R were demanding. Attention is particularly drawn to the sixth inclusion criterion—early relapse following any line of treatment in RR-CLL patients (up to 6 months), early disease progression (6–24 months) after the first line treatment, or with the presence of 17p deletion or *TP53* mutation, the presence of which may have resulted in shorter PFS in the presented analysis as compared to that observed in the MURANO trial. With the highest probability, this criterion seems to have been driven by economic considerations—it served to select a narrow group of patients who, due to their existing burdens, might not have benefited from other forms of treatment. According to the program authors’ assumption, the new type of therapy offered them a chance of recovery. One can argue whether such an approach was correct. The answer may come from the updated drug program introduced lately—patients previously treated with at least one line of therapy are now eligible for therapy, regardless of del17p or *TP53* mutation status. Given the above, it seems economically sensible to broaden the target group so that resources reach those more likely to profit from them. The authors will conceive an evaluation of treatment efficacy in this group in the future.

As mentioned above, the presented RR-CLL group was initially burdened with factors increasing the risk of treatment failure. Therefore, in the study group, the percentage of heavily pretreated and potentially resistant to treatment patients was increased compared to the cohort reported in the MURANO study—in our study, 32 patients (27.2%) had relapsed following the first line of treatment, the number of previous lines of treatment was also significantly higher (median 2, range 1–9). Twenty-two participants were previously treated with BTKi (18.8% out of 117). In contrast, in the MURANO study, only four patients had completed more than three lines of treatment. Our patients were older than those in the MURANO study (median of 67 vs. 65 years) and had a worse performance status—only 10 (9%) patients had an ECOG score of 0. In the MURANO study, however, 57.2% of patients had an ECOG score of 0 [11].

Furthermore, the frequency of adverse events was higher in our analyzed group of patients than in the MURANO study. The most common treatment-related adverse event was neutropenia (74.4% of all patients; grade ≥ 3 in 57.3%), probably associated with the multiplicity of prior treatment lines in the study group. In the MURANO study, treatment was complicated by neutropenia (WHO any grade) in only 60.8% of patients (57.7%; grade 3/4) despite long median exposure to VEN-R therapy. 47.9% of patients in the MURANO study received granulocyte-colony-stimulating factor (G-CSF) as primary and secondary prophylaxis of neutropenia [11]. For our study group, we did not, however, collect data on G-CSF administration.

We also noted a significant number of infections, including pneumonia (23.9% of all patients) and neutropenic fevers (6.8%). In the MURANO study, grade 3 or 4 infectious events occurred in 17.5% of patients, neutropenic fever in 3.6%, and pneumonia in 5.2%. During the follow-up, we observed 20 (17.1%) cases of biochemical tumor lysis syndrome (TLS) and 6 (5.1%) cases of clinical TLS. The rate of grade 3 or 4 tumor lysis syndrome in the VEN-R group in the MURANO trial was 3.1% (6 of 194 patients) [11].

The high percentage (*n* = 21, 17.9%) of patients with a history of high-dose methylprednisolone treatment, which causes significant and often long-term immune suppression, in our study group is noteworthy. Therefore, we investigated this subject and indicated that, among our study group, there was no statistically significant relationship between the occurrence of pneumonia and a history of HDMP in both the SARS-CoV-2 and non-SARS-CoV-2 pneumonia groups. We also evaluated the scenario in which the last line of treatment before VEN-R was HDMP—which also did not affect the occurrence of pneumonia in the study group in this analysis.

During follow-up, 42.7% (*n* = 50/117) of patients were withdrawn from treatment mainly due to the death of a patient (*n* = 15, 30%), therapy-related cytopenias (*n* = 8, 16%), disease progression (*n* = 10, 20%), and infection (*n* = 6, 12%).

In the MURANO study, 5.2% of patients died from treatment complications. In our group, there were 36 deaths reported (30.8%), with up to 30% related to SARS-CoV-2 infection and 8.3% due to other infections. Our results are consistent with our recently published analysis of the incidence of SARS-CoV-2 infection in CLL patients and showed the worse prognosis of CLL patients, regardless of administered therapy [11, 15]. Our results are also consistent with the observations of already published analysis of SARS-CoV-2 infection in CLL patients [16–19]. In our opinion, the inferior results of VEN-R treatment in our group may also be related to the time coincidence of introducing this therapeutic option in Poland and the outbreak of the SARS-CoV-2 pandemic.

The abovementioned differences are characteristic for comparison between registration and real-life studies. We would like to emphasize the impact of the SARS-CoV-2 pandemic, which significantly increased mortality in CLL patients more susceptible to infections than the general population [15–19]. It is plausible that this could significantly account for the increased number of deaths from pneumonia compared with the MURANO study [11]. Important factors influencing the course of the study certainly included its retrospective character, which may have affected especially the process of reporting adverse events due to a possible lack of data on less severe adverse effects of treatment and shorter follow-up time. Additionally, as mentioned before, we did not have data on using G-CSF in Polish patients.

The updated 5-year results from the MURANO trial indicate that with a median follow-up of 59.2 months, the median PFS for VEN-R remains at 53.6 months. However, the contribution of Mato et al.’s study [20], in which the efficacy of CLL VEN monotherapy vs. VEN + anti-CD20 antibody treatment was compared in a group of heavily pretreated patients at higher risk than MURANO patients in terms of both response data and survival outcomes, cannot be overlooked in this study. Although the estimated median PFS and OS were not reached in this study, the estimated 12-month PFS and OS were 74% and 82% for the entire cohort, respectively. This result is consistent with the treatment outcomes in our population (Fig. 1) in which the estimated 12-month PFS was 67.5% and OS was 70%. The above study demonstrated that adding an anti-CD20 antibody in combination with VEN did not impact responses and survival outcomes compared to VEN in monotherapy. The authors then hypothesized that the lack of treatment effect may have been due to the significant number of patients with prior exposure to anti-CD20 antibodies in the cohort. Therefore, overtreated patients do not gain from this form of therapy. This is an interesting finding in relation to our patient group in which more than 90% (91.5%) of patients have a history of rituximab treatment. It can therefore be speculated that perhaps this is another reason for the poorer treatment outcome in our cohort than in that of the MURANO study.

Considering the lower efficacy of VEN-R treatment in the real-life setting in PALG centers, a more detailed analysis aimed at identifying key failure reasons is necessary.

## Conclusion

In this retrospective analysis, the outcomes of treatment with the VEN-R regimen in a real-world setting were worse than those reported in the MURANO trial. It should be noted that in Poland, the introduction of the VEN-R reimbursement coincided with the SARS-CoV-2 pandemic which might have had an impact on the treatment results. In addition, the restrictive eligibility criteria for VEN-R treatment in Poland resulted in the inclusion of patients with more aggressive CLL and/or heavily pretreated patients who were potentially more exposed to therapy complications and resistance to treatment.

## Supplementary Information

Below is the link to the electronic supplementary material.

Supplementary file1 (DOCX 46 KB)

## Data Availability

The data supporting this study's findings are available from the corresponding author, [B.P.], upon request.
