# Supplementary material for: Clinical efficacy and tolerability of venetoclax plus rituximab in patients with relapsed or refractory chronic lymphocytic leukemia—a real-world analysis of the Polish Adult Leukemia Study Group
Source: Ann Hematol. 2023 Jul 1;102(8):2119–26. doi: 10.1007/s00277-023-05304-4 (PMC10344985; doi:10.1007/s00277-023-05304-4)
Supplement: Supplementary file 1 — Supplementary file1 (DOCX 46 KB) [file 277_2023_5304_MOESM1_ESM.docx]

**Supplementary Data**

Table S1. Impact of clinico-pathological parameters in relation to patients overall survival (OS) after venetoclax-rituximab treatment.

| Parameter | Value | Number | OS | HR | 95% CI | p |
| --- | --- | --- | --- | --- | --- | --- |
| Rai classification | 0-2 | 79 | nr | 1.1 | 0.51-2.38 | 0.7957 |
|  | 3-4 | 34 | nr |  |  |  |
| Binet classification | A+B | 80 | nr | 0.88 | 0.36-2.16 | 0.7793 |
|  | C | 20 | nr |  |  |  |
| Sex | Female | 45 | nr | 0.78 | 0.39-1.57 | 0.487 |
|  | Male | 72 | nr |  |  |  |
| Age | ≤65 | 52 | nr | 0.61 | 0.31-1.22 | 0.16 |
|  | >65 | 65 | 30.93 |  |  |  |
| Del17p | Absent | 79 | nr | 0.7 | 0.27-1.84 | 0.15 |
|  | Present | 25 | 25.9 |  |  |  |
| Del11q23 | Absent | 56 | nr | 1.25 | 0.54-2.89 | 0.9 |
|  | Present | 33 | nr |  |  |  |
| Tri12 | Absent | 68 | nr | 3.75 | 0.49-28.13 | 0.75 |
|  | Present | 10 | nr |  |  |  |
| Del13 | Absent | 40 | nr | 0.63 | 0.27-1.47 | 0.19 |
|  | Present | 42 | 30.93 |  |  |  |
| Del17p or *TP53* mutation | Absent | 59 | nr | 0.64 | 0.23-1.76 | 0.14 |
|  | Present | 31 | 25.9 |  |  |  |
| ECOG performance status | 0-1 | 95 | nr | 1 | 0.42-2.43 | 0.99 |
|  | ≥2 | 17 | nr |  |  |  |
| WBC [G/l] | ≤25 | 50 | nr | 0.6 | 0.31-1.18 | 0.13 |
|  | >25 | 67 | nr |  |  |  |
| PLT [G/l] | ≤100 | 46 | nr | 0.73 | 0.37-1.45 | 0.37 |
|  | >100 | 71 | 30.93 |  |  |  |
| HGB [g/dl] | ≤10 | 44 | nr | 1.29 | 0.66-2.51 | 0.44 |
|  | >10 | 73 | nr |  |  |  |
| LDH [U/l] | Normal | 52 | nr | 0.69 | 0.36-1.35 | 0.29 |
|  | >ULN | 65 | nr |  |  |  |
| BMI score | <18.5 | 0 |  |  |  | 0.12 |
|  | 18.5-25 | 52 | nr |  |  |  |
|  | >25 | 42 | nr |  |  |  |
|  | >30 | 19 | 20.8 |  |  |  |
| Serum creatinine [mg/dl] | ≤1.3 | 99 | nr | 0.42 | 0.2-0.87 | 0.01 |
|  | >1.3 | 18 | 21.03 |  |  |  |
| Spleen | Normal | 19 | nr | 1.28 | 0.56-2.93 | 0.64 |
|  | Enlarged | 96 | nr |  |  |  |
| Lymphadenopathy | ≤ 5cm | 62 | nr | 0.86 | 0.45-1.65 | 0.28 |
|  | >5cm | 51 | nr |  |  |  |
| Cumulative Illness Rating Scale score | ≤6 | 59 | nr | 0.84 | 0.43-1.62 | 0.84 |
|  | >6 | 58 | nr |  |  |  |
| Lines of previous therapies | 0-3 | 86 | 30.93 | 1.53 | 0.7-3.37 | 0.29 |
|  | ≥4 | 31 | nr |  |  |  |
| History of autoimmune hemolytic anemia or thrombocytopenia | Absent | 96 | nr | 0.83 | 0.38-1.81 | 0.63 |
|  | Present | 19 | 27.03 |  |  |  |
| History of Fludarabine resistance | Absent | 102 | nr | 1.23 | 0.43-3.48 | 0.69 |
|  | Present | 15 | nr |  |  |  |

BMI - body mass index; ECOG - Eastern Cooperative Oncology Group; HGB - hemoglobin; HR - hazard ratio; LDH - lactate dehydrogenase, nr - not reached; OS - overall survival; PLT - platelets; ULN - upper limit of normal; WBC - white blood count; 95% CI - 95% confidence interval

Table S2. Impact of clinico-pathological parameters in relation to patients progression-free survival (PFS) after venetoclax-rituximab treatment

| Parameter | Value | Number | PFS | HR | 95% CI | p |
| --- | --- | --- | --- | --- | --- | --- |
| Rai classification | 0-2 | 79 | 36.97 | 1.13 | 0.55-2.33 | 0.75 |
|  | 3 and 4 | 34 | nr |  |  |  |
| Binet classification | A+B | 80 | 36.97 | 1.11 | 0.46-2.69 | 0.82 |
|  | C | 20 | nr |  |  |  |
| Sex | Female | 45 | nr | 0.59 | 0.3-1.16 | 0.12 |
|  | Male | 72 | 30.93 |  |  |  |
| Age | ≤65 | 52 | 36.97 | 0.92 | 0.49-1.72 | 0.8 |
|  | >65 | 65 | 30.93 |  |  |  |
| Del17p | Absent | 79 | 36.97 | 0.7 | 0.27-1.84 | 0.4 |
|  | Present | 25 | 25.9 |  |  |  |
| Del11q23 | Absent | 56 | nr | 1.25 | 0.54-2.89 | 0.44 |
|  | Present | 33 | 30.93 |  |  |  |
| Tri12 | Absent | 68 | nr | 3.75 | 0.49-28.13 | 0.58 |
|  | Present | 10 | 36.97 |  |  |  |
| Del13 | Absent | 40 | nr | 0.63 | 0.27-1.47 | 0.28 |
|  | Present | 42 | 30.93 |  |  |  |
| Del17p or *TP53* mutation | Absent | 59 | 36.97 | 0.64 | 0.23-1.76 | 0.51 |
|  | Present | 31 | 25.9 |  |  |  |
| ECOG performance status | 0-1 | 95 | nr | 0.84 | 0.37-1.93 | 0.69 |
|  | ≥2 | 17 | 36.97 |  |  |  |
| WBC [G/l] | ≤25 | 50 | nr | 0.596 | 0.32-1.12 | 0.1 |
|  | >25 | 67 | 30.93 |  |  |  |
| PLT [G/l] | ≤100 | 46 | 36.97 | 0.89 | 0.47-1.66 | 0.7 |
|  | >100 | 71 | 30.93 |  |  |  |
| HGB [g/dl] | ≤10 | 44 | 36.97 | 1.39 | 0.74-2.6 | 0.3 |
|  | >10 | 73 | nr |  |  |  |
| LDH [U/l] | Normal | 52 | nr | 0.88 | 0.47-1.64 | 0.67 |
|  | >ULN | 65 | 36.97 |  |  |  |
| BMI score | <18,5 | 0 |  |  |  | 0.22 |
|  | 18.5-25 | 52 | 36.97 |  |  |  |
|  | >25 | 42 | nr |  |  |  |
|  | >30 | 19 | 20.8 |  |  |  |
| Serum creatinine [mg/dl] | ≤ 1,3 | 99 | 36.97 | 0.55 | 0.27-1.13 | 0.1 |
|  | >1,3 | 18 | 23.8 |  |  |  |
| Spleen | Normal | 19 | nr | 1.16 | 0.51-2.64 | 0.72 |
|  | Enlarged | 96 | 36.97 |  |  |  |
| Lymphadenopathy | ≤5cm | 62 | 30.93 | 0.76 | 0.41-1.42 | 0.39 |
|  | >5cm | 51 | 36.97 |  |  |  |
| Cumulative Illness Rating Scale score | ≤6 | 59 | 36.97 | 0.85 | 0.45-1.58 | 0.6 |
|  | >6 | 58 | 30.93 |  |  |  |
| Lines of previous therapies | 0-3 | 86 | 30.93 | 1.16 | 0.58-2.33 | 0.67 |
|  | ≥4 | 31 | 36.97 |  |  |  |
| History of autoimmune hemolytic anemia or thrombocytopenia | Absent | 96 | nr | 0.74 | 0.36-1.54 | 0.42 |
|  | Present | 19 | 27.03 |  |  |  |
| History of Fludarabine resistance | Absent | 102 | 36.97 | 0.75 | 0.32-1.8 | 0.52 |
|  | Present | 15 | nr |  |  |  |

BMI - body mass index; ECOG - Eastern Cooperative Oncology Group; HGB - hemoglobin; HR - hazard ratio; LDH - lactate dehydrogenase, nr - not reached; PLT - platelets; PFS - progression-free survival; ULN - upper limit of normal; WBC - white blood count; 95% CI - 95% confidence interval

Table S3. Impact of clinico-pathological parameters in relation to patients’ response to venetoclax-rituximab treatment. Data were analyzed using Fischer exact test.

| Parameter | CR+PR | | SD+NR+PD | |  |
| --- | --- | --- | --- | --- | --- |
|  | n | (%) | n | (%) | p-value |
| Sex | | | | | |
| Female | 42 | 100.0% | 0 | 0.00% | 0.15 |
| Male | 59 | 92.19% | 5 | 7.81% |  |
| Rai classification | | | | | |
| 0-2 | 69 | 95.83% | 3 | 4.17% | 0.63 |
| 3-4 | 28 | 93.33% | 2 | 6.67% |  |
| Binet classification | | | | | |
| A+B | 70 | 95.89% | 3 | 4.11% | 0.55 |
| C | 15 | 93.75% | 1 | 6.25% |  |
| Del17p | | | | | |
| Absent | 67 | 93.06% | 5 | 6.94% | 0.19 |
| Present | 23 | 100.00% | 0 | 0.00% |  |
| Del11q23 | | | | | |
| Absent | 49 | 96.08 % | 2 | 3.92% | 0.35 |
| Present | 27 | 90.00% | 3 | 10.00% |  |
| *TP53* mutation | | | | | |
| Absent | 55 | 93.22% | 4 | 6.78% | >0.99 |
| Present | 12 | 100.00% | 0 | 0.00% |  |
| Lines of previous therapies | | | | | |
| 0-3 | 73 | 94.81% | 4 | 5.19% | >0.99 |
| ≥4 | 28 | 96.55% | 1 | 3.45% |  |
| ECOG performance status | | | | | |
| 0-1 | 84 | 96.55% | 3 | 3.45% | 0.16 |
| ≥2 | 13 | 86.67% | 2 | 13.33% |  |
| WBC [G/l] | | | | | |
| ≤25 | 44 | 95.65% | 2 | 4.35% | >0.99 |
| >25 | 57 | 95.00% | 3 | 5.00% |  |
| PLT [G/l] | | | | | |
| ≤ 100 | 40 | 93.02% | 3 | 6.98% | 0.39 |
| >100 | 61 | 96.83% | 2 | 3.17% |  |
| HGB [g/dl] | | | | | |
| ≤ 10 | 38 | 97.44% | 1 | 2.56% | 0.65 |
| >10 | 63 | 94.03% | 4 | 5.97% |  |
| LDH [U/l] | | | | | |
| Normal | 43 | 91.49% | 4 | 8.51% | 0.17 |
| >ULN | 58 | 98.31% | 1 | 1.69% |  |
| BMI score | | | | | |
| 18.5-25 | 42 | 93.33% | 3 | 6.67% | 0.67 |
| 25-30 | 38 | 97.44% | 1 | 2.56% |  |
| >30 | 17 | 94.44% | 1 | 5.56% |  |
| Serum creatinine [mg/dl] | | | | | |
| ≤1.3 | 86 | 95.56% | 4 | 4.44% | 0.57 |
| >1.3 | 15 | 93.75% | 1 | 6.25% |  |
| History of autoimmune hemolytic anemia or thrombocytopenia | | | | | |
| Absent | 83 | 95.40% | 4 | 4.60% | >0.99 |
| Present | 16 | 94.12% | 1 | 5.88% |  |
| Age [years] | | | | | |
| ≤65 | 45 | 91.84% | 4 | 8.16% | 0.18 |
| >65 | 56 | 98.25% | 1 | 1.75% |  |
| Tri12 | | | | | |
| Absent | 60 | 93.75% | 4 | 6.25% | >0.99 |
| Present | 10 | 100.00% | 0 | 0.00% |  |
| Del13 | | | | | |
| Absent | 36 | 92.31% | 3 | 7.69% | 0.62 |
| Present | 36 | 97.30% | 1 | 2.70% |  |
| Cumulative Illness Rating Scale score | | | | | |
| 0-6 | 50 | 94.34% | 3 | 5.66% | >0.99 |
| >6 | 51 | 96.23% | 2 | 3.77% |  |
| History of fludarabine resistance | | | | | |
| Absent | 88 | 96.70% | 3 | 3.30% | 0.15 |
| Present | 13 | 86.67% | 2 | 13.33% |  |
| Lymphadenopathy | | | | | |
| Absent | 56 | 94.92% | 3 | 5.08% | >0.99 |
| Present | 42 | 95.45% | 2 | 4.55% |  |
| Spleen | | | | | |
| Normal | 17 | 94.44% | 1 | 5.56% | 0.54 |
| Enlarged | 83 | 96.51% | 3 | 3.49% |  |
| Del17p or *TP53* mutation | | | | | |
| Absent | 50 | 92.59% | 4 | 7.41% | 0.29 |
| Present | 29 | 100.0% | 0 | 0.00% |  |

BMI - body mass index; CR - complete remission; ECOG - Eastern Cooperative Oncology Group; HGB - hemoglobin; HR - hazard ratio; LDH - lactate dehydrogenase, ND - no data; NR - no remission; PD - progressive disease; PFS: progression-free survival; PLT: platelets; PR: partial remission; SD: stable disease; ULN: upper limit of normal; WBC: white blood count; 95% CI: 95% confidence interval.
